# Osteosarcopenia: epidemiology, diagnosis, and treatment—facts and numbers

**DOI:** 10.1002/jcsm.12567

**Published:** 2020-03-22

**Authors:** Ben Kirk, Jesse Zanker, Gustavo Duque

**Affiliations:** ^1^ Department of Medicine, Western Health, Melbourne Medical School University of Melbourne Melbourne Australia; ^2^ Australian Institute for Musculoskeletal Science (AIMSS) University of Melbourne and Western Health Melbourne Australia

**Keywords:** Osteosarcopenia, Bone, Muscle, Falls, Fractures, Mortality

## Abstract

**Background:**

Osteosarcopenia, the presence of osteopenia/osteoporosis and sarcopenia, is an emerging geriatric giant, which poses a serious global health burden.

**Methods and results:**

The prevalence of osteosarcopenia ranges in community‐dwelling older adults [5**–**37% (≥65 years)] with the highest rates observed in those with fractures (low‐trauma fracture: ~46%; hip fracture: 17.1**–**96.3%). Among 2353 community‐dwelling adults, risk factors associated with osteosarcopenia include older age [men: 14.3% (60**–**64 years) to 59.4% (≥75 years); women: 20.3% (60**–**64 years) to 48.3% (≥75 years), *P* < 0.05], physical inactivity [inverse relationship: 0.64, 95% confidence interval (CI) 0.46–0.88 (sexes combined)], low body mass index (inverse relationship: men: 0.84, 95% CI 0.81–0.88; women: 0.77, 95% CI 0.74–0.80), and higher fat mass (men: 1.46, 95% CI 1.11–1.92; women: 2.25, 95% CI 1.71–2.95). Among 148 geriatric inpatients, osteosarcopenic individuals demonstrate poorer nutritional status (mini‐nutritional assessment scores: 8.50 ± 2.52 points, *P* < 0.001) vs. osteoporosis or sarcopenia alone, while among 253 older Australians, osteosarcopenia is associated with impaired balance and functional capacity [odds ratios (ORs): 2.56**–**7.19; *P* < 0.05] vs. non‐osteosarcopenia. Osteosarcopenia also associates with falls (ORs: 2.83**–**3.63; *P* < 0.05), fractures (ORs: 3.86**–**4.38; *P* < 0.05), and earlier death [hazard ratio (1‐year follow‐up): 1.84, 95% CI; 0.69**–**4.92, *P* = 0.023] vs. non‐osteosarcopenia.

**Conclusions:**

This syndrome is expected to grow in age‐related and disease‐related states, a likely consequence of immunosenescence coinciding with increased sedentarism, obesity, and fat infiltration of muscle and bone. Evidence suggests the pathophysiology of osteosarcopenia includes genetic polymorphisms, reduced mechanical loading, and impaired endocrine functioning, as well as altered crosstalk between muscle, bone, and fat cells. Clinicians should screen for osteosarcopenia via imaging methods (i.e. dual‐energy X‐ray absorptiometry) to quantify muscle and bone mass, in addition to assessing muscle strength (i.e. grip strength) and functional capacity (i.e. gait speed). A comprehensive geriatric assessment, including medical history and risk factors, must also be undertaken. Treatment of this syndrome should include osteoporotic drugs [bone anabolics/antiresorptives (i.e. teriparatide, denosumab, bisphosphates)] where indicated, and progressive resistance and balance exercises (at least 2‐3 times/week). To maximize musculoskeletal health, nutritional recommendations [protein (1.2**–**1.5 g/kg/day), vitamin D (800–1000 IU/day), calcium (1300 mg/day), and creatine (3**–**5 g/day)] must also be met. It is anticipated that diagnosis and treatment for osteosarcopenia will become part of routine healthcare in the future. However, further work is required to identify biomarkers, which, in turn, may increase diagnosis, risk stratification, and targeted treatments to improve health outcomes.

## Introduction

Healthy aging depends on the ability to maintain the reserve capacity of multiple physiological systems. Of those, the musculoskeletal (MSK) system not only enables human ambulation but also serves as a major metabolic storage site (i.e. acts as a reservoir for calcium in bone as well as glucose in muscle). However, as an older person reaches their sixth decade of life, there is a progressive decline in bone mineral density (BMD) (~1**–**1.5% per year) and muscle mass (~1% per year) and strength (~2.5**–**3% per year),^1^, ^2^ which predisposes to the risk of osteoporosis and sarcopenia—two diseases with medical classifications listed by the International Classification of Diseases.

The World Health Organization defines osteopenia and osteoporosis as a T score equal to or less than −1 and −2.5 standard deviations, respectively, below the peak bone mass of a young healthy cohort or in the presence of a minimal‐trauma fracture.^3^ This skeletal disease reduces bone microarchitecture and impedes bone strength.^3^ On the other hand, sarcopenia is characterized by cut‐off values for low muscle mass, strength, and/or functional capacity^4^ and associates with a range of metabolic conditions.^5^ Both diseases share common risk factors^3^, ^6^ and are strongly associated with frailty, falls, fractures, hospitalizations, and mortality,^7^, ^8^, ^9^ as well as causing an upsurge in healthcare expenditure.

In 2010 alone, there was a respective 5.5 and 22 million men and women living with osteoporosis in the European Union, resulting in roughly 3.5 million fragility fractures and costing over €37 billion,^10^ a figure that is projected to increase by 25% in 2025. Likewise, using longitudinal data from the Hertfordshire trial, muscle weakness (characterized by low grip strength) was associated with an annual cost of £2,707 per person in the UK, with an overall estimated cost of £2.5 billion in 2018.^11^ Alarmingly, the aging population is now ‘moving less and eating more’ in community‐dwelling and aged‐care facilities, enabling a trend towards an MSK phenotype with low bone and muscle mass and increased ectopic fat, which may manifest as osteosarcopenia.^6^

Osteosarcopenia was first coined by Duque and colleagues^6^ to describe a subset of older persons affected by osteoporosis and sarcopenia. It is important to note that osteosarcopenia is a unique syndrome, defined by the combination of low bone density (osteopenia/osteoporosis) and muscle mass, strength, and/or functional capacity (sarcopenia).^3^ As a consequence of an aging population, which will see an increase in older persons (≥60 years) from ~841 million in 2013 to ~2 billion by 2050 (proportional increase of 9%),^8^ the prevalence of osteosarcopenia will inevitably increase, resulting in a greater number of falls, fractures, and hospitalizations. This article aims to increase the awareness of an underappreciated MSK syndrome by providing clinicians with an overview of the epidemiology, pathophysiology, diagnosis, and treatments for osteosarcopenia.

### Osteoporosis and sarcopenia: osteosarcopenia

Muscle and bone loss often coincides in older persons, and a plethora of studies has demonstrate a strong relationship between the components (osteoporosis and sarcopenia) of osteosarcopenia.^12^ In a cohort of 590 Finnish post‐menopausal women, those with sarcopenia possessed a 12.9 times higher risk [95% confidence interval (CI) 3.1–53.5] of having osteoporosis vs. those without sarcopenia.^13^ Among the Sarcophage Cohort of 232 older persons, those with sarcopenia had a fivefold higher risk of developing osteoporosis (95% CI 1.16–19.41). Two subsequent cross‐sectional^14^, ^15^ and one longitudinal study^16^ showed that osteoporosis strongly increases the risk of sarcopenia and vice versa. A very recent study among 3334 older adults demonstrated that individuals with probable and confirmed sarcopenia (compared with no sarcopenia) had lower BMD and bone architecture at various anatomical sites when employing the 2019 European definition of sarcopenia.^17^ As seen, a bidirectional relationship exists between osteoporosis and sarcopenia, which leads to the development of osteosarcopenia.

## Pathophysiology

A myriad of factors may explain the pathology of osteosarcopenia. First, polymorphisms of the genes glycine‐*N*‐acyltransferase (GLYAT), methyltransferase like 21C (METTL21C), peroxisome proliferator‐activated receptor gamma coactivator 1‐alpha (PGC‐1α), and myocyte enhancer factor‐2 (MEF2C) associate with muscle atrophy and bone loss.^3^, ^6^ In addition, genetic traits determine peak muscle and bone volume in early life,^3^, ^6^ which may be a mechanism for delaying sarcopenia and osteoporosis in late life. Second, gravitational loading (via external ground forces or internal muscle contractions) is transferred from muscle to the skeleton, providing the mechanical stimuli to maintain bone density.^3^, ^6^ Indeed, physical inactivity common in old age or in states of disuse (bed rest, hip fracture) results in atrophy of both tissues,^3^, ^5^ while physical loading is hypertrophic to muscle and osteogenic to bone.^18^ Third, the metabolism of both tissues is similar in that amino acid availability determines the rate of protein turnover in muscle while contributing to the bone matrix by enabling collagen synthesis.^19^ With aging, the sensitivity of the MSK system to utilize dietary protein and vitamin D deteriorates and may be an overlapping risk factor resulting in joint catabolism.^3^ These nutrients also regulate cellular proteins and growth factors via the release of insulin‐like growth factor 1, inhibition of parathyroid hormone, and facilitating calcium uptake, all involved in muscle and bone kinetics.^19^ Lastly, across the life cycle, hormonal factors are implicated in osteosarcopenia. For instance, low testosterone and estrogen are adversely associated with muscle atrophy and bone loss in men and women, respectively.^3^, ^6^ A plethora of work from animal and human models also shows that low concentrations of growth hormone and its derivative insulin‐like growth factor 1 associate with impaired bone remodelling and muscle protein turnover.^20^

While the aforementioned genetic, mechanical, and endocrine factors may, in part, explain the age‐related association between muscle and bone loss, there is accumulating evidence that other localized and systemic factors are involved. Indeed, mesenchymal stem cells residing in connective tissue (muscle, bone, and fat) are implicated in osteosarcopenia.^6^ For instance, muscle‐derived myokines such as myostatin, follistatin, and irisin have direct effects on bone remodelling, with the former inducing osteoclastogenesis while the latter two inhibit bone resorption.^3^ In the opposite direction, osteocalcin and connexin 43, bone‐derived osteokines, have modulating effects on muscle anabolism and catabolism, receptively.^6^ Finally, aging is linked with an accumulation of intramuscular and bone marrow fat, which secretes adipokines known to induce apoptosis of myocytes and osteocytes.^21^ We have recently shown that the fatty acid palmitic acid is highly expressed in aged muscle and bone and creates a lipotoxic environment to the surrounding tissue.^22^

**Figure 1:**
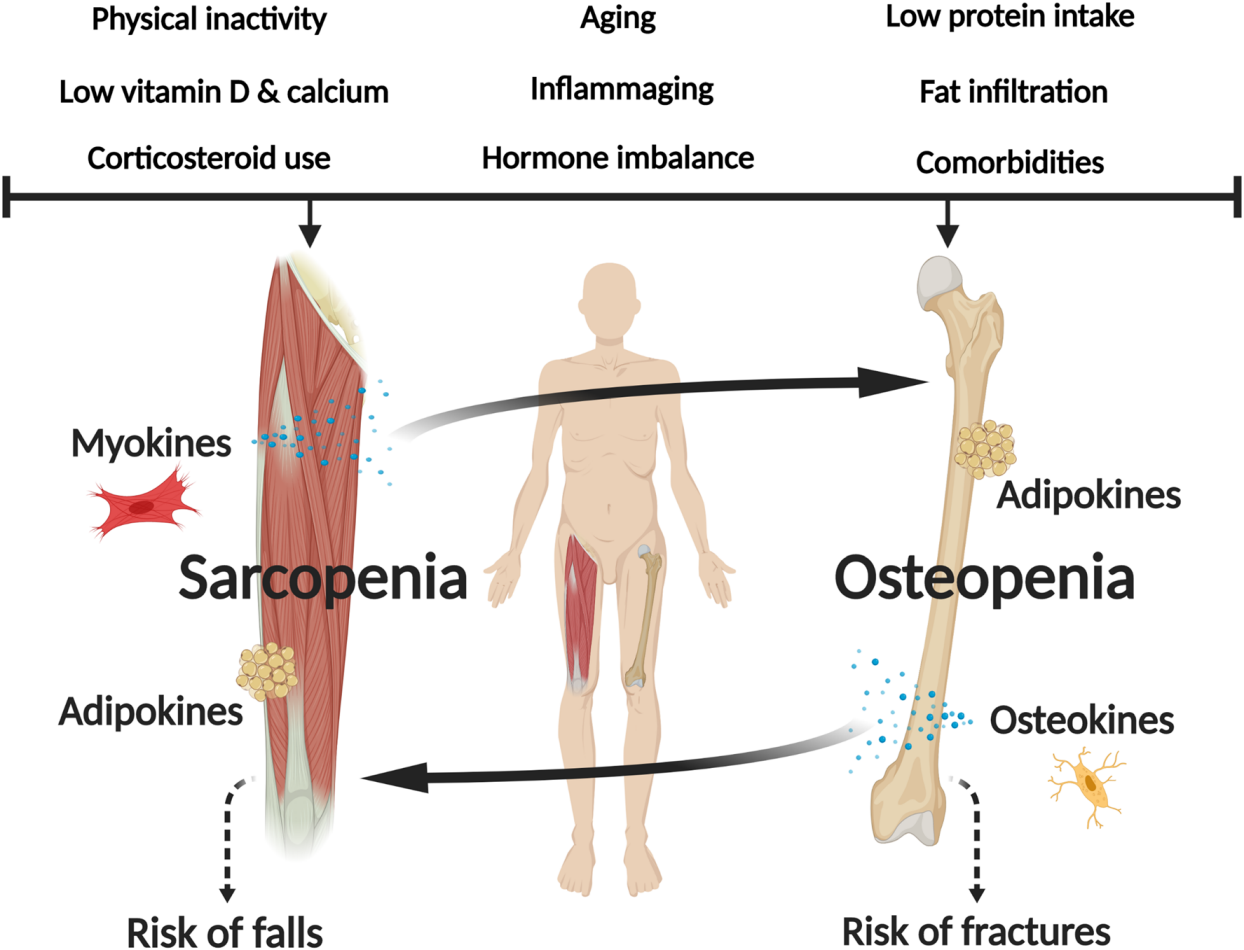
Risk factors, muscle–bone crosstalk (myokines, osteokines, adipokines), and the pathophysiology of osteosarcopenia.

As seen, the pathophysiology underpinning osteosarcopenia is only emerging, although numerous catabolic factors driven by immunosenescence have already shown to play a bidirectional role in muscle and bone (*Figure* 1).

## Epidemiology

### Prevalence

Among community‐dwelling populations, the prevalence of osteosarcopenia increases with age [men: 14.3% (60**–**64 years) to 59.4% (≥75 years); women: 20.3% (60**–**64 years) to 48.3% (≥75 years), *P* < 0.05]^23^ and is greater in women (2.5.5–82.6%) than men (16.4**–**32.0%).^12^ Older persons with a minimal‐trauma fracture (~46%) or post‐hip fracture (17.1**–**96.3%) demonstrate the highest prevalence rates of osteosarcopenia,^12^ which represents a critical manifestation of aging and multimorbidity. This variance in prevalence estimates for ostensibly similar populations reflects significant misclassification due to the differences in definitions for sarcopenia; the definition for osteopenia/osteoporosis is consistent worldwide.

### Risk factors

As highlighted above, common risk factors for osteosarcopenia are age and sex. A recent population‐based study of 2353 community‐dwelling older adults also found that body mass index (men: 0.84, 95% CI 0.81–0.88; women: 0.77, 95% CI 0.74–0.80) and physical activity [0.64, 95% CI 0.46–0.88 (sexes combined)] were inversely associated with osteosarcopenia, while higher fat mass increased the risk (men: 1.46, 95% CI 1.11–1.92; women: 2.25, 95% CI 1.71–2.95).^23^ Each year of schooling was also associated with a 3% lower prevalence of osteosarcopenia in men (0.97, 95% CI 0.95–0.99).^23^ In other study among 148 geriatric inpatients, osteosarcopenic individuals were at greater risk of malnourishment (mini‐nutritional assessment scores: 8.50 ± 2.52 points, *P* < 0.001)^24^ compared with osteoporosis or sarcopenia alone, and among 253 older Australians, osteosarcopenia is associated with poorer balance and functional capacity [odds ratios (ORs) ranging from 2.56 to 7.19; *P* < 0.05] vs. osteopenia and osteoporosis alone.^7^ Others have noted that muscle strength and functional performance measures are also lower in those with osteosarcopenia vs. osteoporosis or sarcopenia alone.^17^, ^25^

### Clinical outcomes

When compared to non‐osteosarcopenic individuals, the risk of falls (ORs: 2.83–3.63; P < 0.05) and fractures (ORs: 3.86–4.38; P < 0.05) when using multiple sarcopenia definitions is significantly higher in osteosarcopenic older adults attending a falls and fractures clinic.^7^

The risk of incurring a minimal‐trauma or no trauma fracture when sarcopenic was also found to be much greater than in non‐sarcopenic older persons (relative risk 1.37, 95% CI 1.18–1.58).^12^ A recent meta‐analysis corroborates this finding with the odds of a fracture in sarcopenic compared with non‐sarcopenia older persons reported as 1.84 (95% CI 1.30–2.62).^26^In those with hip fractures, the risk of mortality is also higher in 93 osteosarcopenic patients [hazard ratio (1‐year follow‐up): 1.84, 95% CI; 0.69–4.92, P = 0.023] vs. non‐osteosarcopenic patients.^9^ In contrast to these findings, two longitudinal studies in Australian men did not show an increased risk of falls, fractures, or mortality beyond the effect of osteoporosis or sarcopenia alone.^27^, ^28^ The heterogeneity in findings between studies likely relates to inconsistent use of sarcopenia definitions. In addition, most of these studies utilized dual‐energy X‐ray absorptiometry (DXA)‐derived estimates of muscle mass, which is confounded by other organ masses and connective tissues. Further longitudinal trials are needed to determine whether osteosarcopenic individuals are at an increased risk of falls, fractures, and earlier death when compared with sarcopenia or osteoporosis alone. These trials should utilize direct measures of muscle mass such as the creatine dilution method, which has shown to be a strong predictor of falls and fractures while DXA‐derived estimates of muscle mass was not.^29^ [Correction added on 16 April 2020 after first online publication: The first and fourth sentences have been corrected in this current version.]

### Clinical assessment

**Figure 2:**
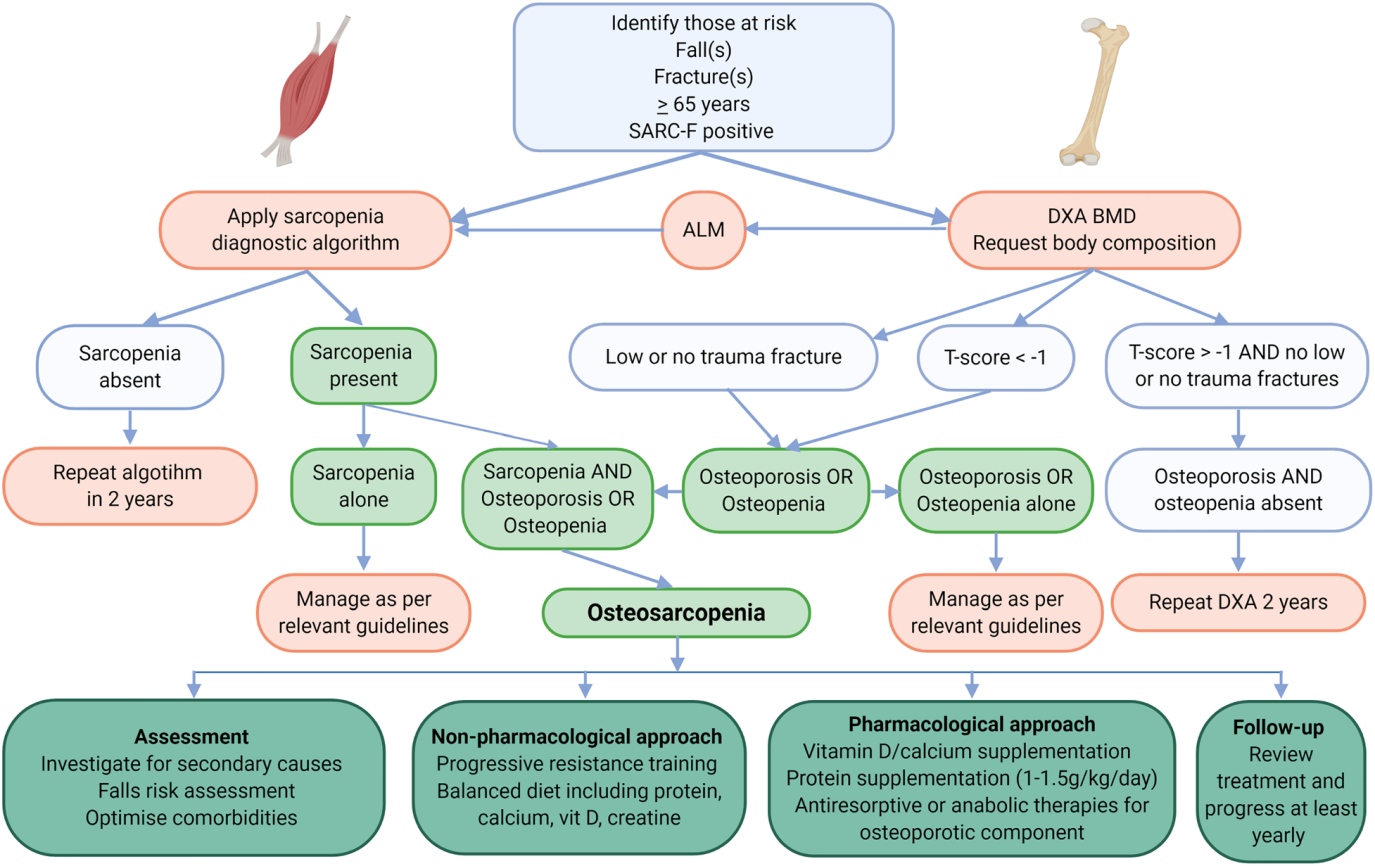
Clinical algorithm to assess and manage osteosarcopenia. ALM, appendicular lean mass; BMD, bone mineral density; DXA, dual‐energy X‐ray absorptiometry.

Individually, osteoporosis and sarcopenia remain underdetected and undertreated. There are strong recommendations for active case finding for both osteoporosis/osteopenia and sarcopenia.^4^ The identification of either condition should prompt investigation for osteosarcopenia given the high rate of co‐occurrence of osteoporosis/osteopenia and sarcopenia in older adults.^4^ We have previously argued that osteosarcopenia should be considered an integral component of the comprehensive geriatric assessment.^30^ Indeed, the assessment for osteosarcopenia involves a thorough history (including medical, social, falls, fractures, and medications histories), risk factor identification, physical assessments, and targeted investigations.^30^ *Figure* 2 outlines an approach that clinicians may adopt in assessing and managing older adults at risk of osteosarcopenia.

### History, screening, and risk factor identification

A comprehensive history allows the clinician to judiciously determine the risk, causes, and implications of osteosarcopenia and will inform person‐centred treatment recommendations. Given the high rates of sensory and cognitive impairments in persons most at risk of osteosarcopenia, collateral history from family, next of kin, carers, and health professionals may be required. There is significant overlap between the putative causes of osteoporosis/osteopenia and sarcopenia, and together, these may be considered primary or secondary. Primary causes may be age related, occurring in the absence of any recognized secondary cause. Secondary causes may be related to concomitant disease, activity, malnutrition, and medications (*Table* 1). Further, clinicians should complete a thorough falls history particularly examining for and intervening to address modifiable falls risk factors.

**Table 1:** Secondary causes of osteosarcopenia

Disease related	Activity related	Nutrition and medication related
*Endocrine disease* Type II diabetes mellitus, hypogonadism, early menopause, thyroid disorders, hypercalciuria, Paget's disease, cortisol excess, hypogonadism *Inflammatory disease* Rheumatoid arthritis*Malignant disease* Cancer (solid organ and blood based)*Organ failure* Failures of heart, lung, liver, kidney, or brain	Bedridden state Hospitalization Institutionalization Prolonged weightlessness Sedentary lifestyle Socioeconomic status	*Nutrition* Alcohol excess, cachexia, low body weight, low protein intake, low fat‐soluble vitamin intake, malabsorptive conditions, smoking *Medications* Glucocorticoid therapy, chemotherapeutics, heparin, antiepileptics, aromatase inhibitors, GnRH agonists, excess thyroxine

GnRH, gonadotrophin‐releasing hormone

There are no screening or risk calculation tools validated for osteosarcopenia. However, numerous tools are at the clinician's disposal for both osteoporosis and sarcopenia. The SARC‐F is a 5‐point sarcopenia questionnaire recommended in the most recent international consensus guidelines.^31^ Owing to its moderate sensitivity and high specificity, the SARC‐F is most accurate in detecting those with severe sarcopenia. The SARC‐F has been validated in international and multiethnic populations.^31^ In contrast, there is clear consensus on osteoporosis screening and when investigations with BMD testing via DXA should be undertaken. BMD should be considered in all adults aged over 50 at risk of or with a previous fracture, post‐menopausal women, men over the age of 70, or adults with a condition (e.g. rheumatoid arthritis) or medication (e.g. corticosteroids) known to cause bone loss.^30^ There are seven validated tools for risk stratification in those with osteoporosis; however, the FRAX^©^ is most widely used and cited.^32^ The FRAX^©^ can be applied in the absence of BMD (such as in resource‐poor settings) and has been validated across 80% of global populations.^30^

### Physical assessment

A physical examination should be routine in the comprehensive geriatric assessment. However, additional physical assessments are required to diagnose sarcopenia. Physical assessments are considered as either measures of muscle strength (grip strength, sit to stand test) or functional capacity (gait speed, short physical performance battery, timed up and go test, 400 m walk test). The two most widely used and validated assessments are grip strength and gait speed.^31^ Clinicians must apply caution when using these measures interchangeably; different strength and performance measures result in markedly different classifications of sarcopenia within populations and individuals.^33^

### Investigations

Targeted investigations addressing modifiable risk factors identified in the history and physical assessment may be required based on clinician suspicion. Most secondary causes of pathology leading to the increased risk of falls and fractures can be detected by testing the serum for 25(OH) vitamin D, calcium, parathyroid hormone, and serum testosterone (in men).^34^ However, certain investigations are required for osteosarcopenia to make the diagnosis and inform management decisions.

Muscle mass, quantity or quality, and BMD are the focus of investigations in the workup of osteosarcopenia. Multiple tools and techniques are available to clinicians and researchers in order to characterize and quantify muscle and bone. DXA is the most commonly used tool in research and clinical practice to accurately determine BMD including response to osteoporosis treatment. DXA has the dual advantage of providing an accurate estimate of lean body mass, and appendicular lean mass (ALM) is correlated with (but overestimates) muscle mass.^35^ ALM [with adjustments for either body mass index (kg/m^2^) or height^2^ (m)] is a component of the most recent sarcopenia definitions and clinical practice guidelines.^31^ However, the value of ALM being included in future sarcopenia definitions has been questioned, particularly considering its lack of independent association with some negative outcomes in older adults.^36^

Other techniques used in the assessment of muscle quality or quantity include bioelectrical impedance analysis (estimates fat‐free mass), peripheral quantitative computerized tomography, which estimates bone structure and muscle cross‐sectional area and intramuscular adipose tissues, and magnetic resonance imaging (measures small muscle volume.). A novel technique for measuring muscle mass, the D_3_‐creatine dilution method, has recently shown strong relation with falls, fracture, and mortality risk in older men.^35^ This technique requires further validation in different populations before being considered in routine clinical care.

The indications for BMD testing with DXA are described above. Alternative techniques to DXA that estimate BMD include peripheral DXA, quantitative computerized tomography, quantitative ultrasound, and radiographic absorptiometry. Due to population distribution, most older adults who experience a low‐trauma fracture have BMD in the normal or osteopenic range. BMD assessment techniques have high sensitivity and low specificity for fracture prediction.^30^

## Treatment: progressive resistance and balance exercises and adequate nutrition

**Figure 3:**
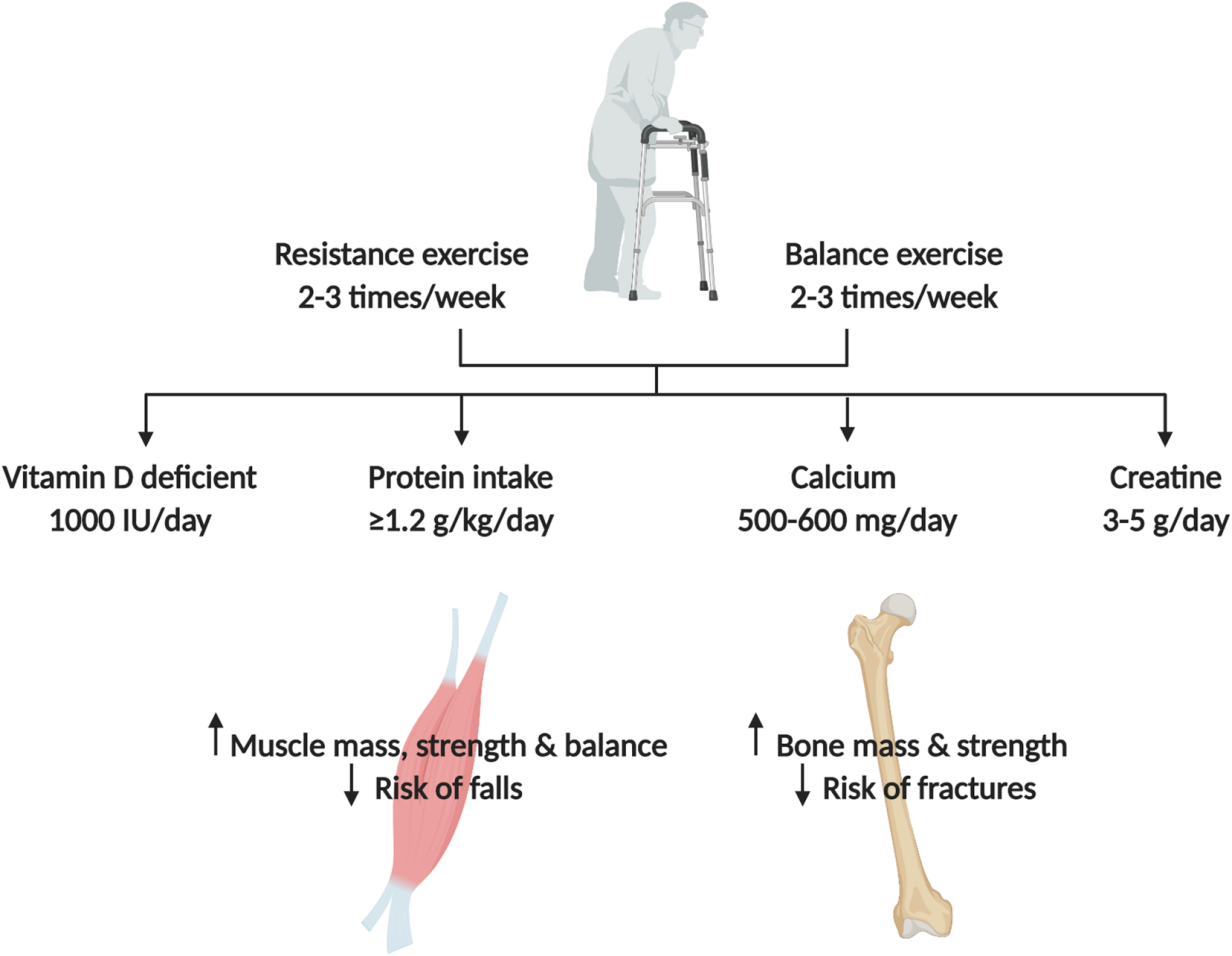
Lifestyle treatments for osteosarcopenia.

Randomized controlled trials (RCTs) have demonstrated the efficacy of progressive resistance exercise to stimulate osteoblastogenesis and muscle protein synthesis, leading to improvements in bone microarchitecture, muscle mass, strength, and functional capacity in osteoporotic and sarcopenic older adults.^18^, ^37^, ^38^ However, the benefits of resistance exercise are not exclusive to the MSK system alone, with positive adaptations on endothelial, myocardial, and cognitive functioning also occurring.^39^ We recommend this mode of exercise to prevent osteosarcopenia and other chronic diseases associated with aging (*Figure* 3).

Regarding the delivery of nutrients, it is well established that the sensitivity of the MSK system to utilize dietary protein and its constituent amino acids deteriorates. As such, RCTs have examined the effect of protein supplementation (above the recommended daily amount 0.8 g/kg/day) in conjunction with resistance exercise interventions and demonstrated augmentations in muscle and bone mass, as well as muscle strength, balance, and functional capacity.^38^, ^40^ Whey protein, a fast‐digesting and fast‐absorbing protein, contains abundant levels of leucine (a key stimulator of mTORC1 in skeletal muscle), and is the most potent dietary strategy to increase muscle protein synthesis.^41^ However, increasing protein intake is more effective when vitamin D levels are in an optimal range.^42^ Supplementation with at least 1000 IU/day of vitamin D may be needed to achieve this target while protecting bone health, as the bioavailability of this micronutrient deteriorates in geriatric patients. Of notice, 13 weeks of a nutritional beverage (consisting of vitamin D and leucine‐enriched whey protein) increased appendicular lean (muscle) mass and chair‐stand speed^43^ and attenuated markers of inflammation^44^ in sarcopenic older adults. However, a larger study showed no benefit of whey protein supplementation on curbing declines in muscle mass and physical function in sarcopenic older adults,^45^ although compliance with the supplement was ~58%, which corroborates another study^38^ displaying the difficulties of supplementing in this population. Irrespective of this, expert consensus groups recommend at least 1.2–1.5 g/kg/day (with 2.5–3 g of leucine per meal) for older adults to promote the accretion of muscle contractile proteins.^46^ Another option is the leucine metabolite β‐hydroxy β‐methylbutyrate, which is also effective at stimulating muscle protein synthesis and attenuating muscle catabolism,^41^ although further RCTs are needed to demonstrate its efficacy in osteosarcopenic individuals.

Calcium is the most abundant mineral in bone, and findings from animal models suggest a role of this nutrient in facilitating muscle contractile force via the maintenance of calcium kinetics.^47^ Although the benefits of calcium in reducing fracture risk are equivocal, reference guidelines suggest an intake of 1000–1300 mg/day which should be met through supplementation if dietary intake is suboptimal.^47^ Despite the recent controversy relating to the proposed risk of calcium supplementation, the most recent cross‐sectional study from UK Biobank showed no association with all‐cause mortality.^48^

Finally, creatine has consistently shown to augment exercise‐induced increases in muscle mass and strength,^49^ and recent reports suggest that this nutrient may increase bone density.^49^ Further research is needed to clarify the effects of creatine monohydrate in osteosarcopenic populations, particularly in respect to adaptations in bone microarchitecture using high‐resolution imaging.

## Pharmacological advancements

At present, there are no Food and Drug Administration‐approved pharmacological agents for sarcopenia, which may reflect the novelty of sarcopenia as a recently established condition. In contrast, pharmacotherapy for osteoporosis is widely available. Therapies include antiresorptive (denosumab, bisphosphonates), anabolic (teriparatide, abaloparatide), antisclerostin (romosozumab), and hormonal (hormone replacement therapy, selective oestrogen receptor modulators) agents. The indications, cost, availability, and approval of these different agents vary globally, and we have summarized these elsewhere.^50^ Those who benefit from antiresorptive or anabolic treatment of osteoporosis, according to the National Osteoporosis Foundation, include adults with a minimal‐trauma hip or vertebral fracture; a T score of −2.5 or less on DXA, or a FRAX^©^ 10‐year fracture risk of ≥3% at the hip or ≥20% for any other osteoporotic fracture.^51^ Prior to treatment, persons must be vitamin D replete (>50 nmol/L preferred) and be counselled on the risks and potential adverse effects of the agents.^51^

Pharmacologic therapies specifically treating osteosarcopenia have not yet been developed although one agent, denosumab, a RANK ligand inhibitor, has shown promising effects on muscle and bone. In one trial, denosumab was compared with either zoledronic acid or alendronate in women with sarcopenia over a 3‐year period.^52^ Those receiving denosumab experienced significant increases in handgrip strength and lean body mass while treatment with bisphosphonate resulted in no change in these measures.^52^ More recently, in a non‐randomized study of community‐dwelling older adults attending a falls and fracture clinic, denosumab treatment improved balance, fear of falling, and physical function, whereas zoledronic acid did not.^53^ These results are promising; however, further double‐blind RCTs are needed to confirm these findings and to determine the impact of denosumab on falls and fractures in osteosarcopenic patients.

### Follow‐up

Those in whom a diagnosis of osteosarcopenia is made require ongoing monitoring including reassessment of falls and fracture risk, quality of life impact, and treatment response. As outlined in *Figure* 2, a clinician review should be undertaken at least yearly (or more frequently if there are changes in clinical circumstance). In those who remain at risk (e.g. with osteoporos/osteopenia or sarcopenia alone, 65 years and over, or with falls), we argue that the diagnostic algorithm should be repeated twice per year or sooner if clinically indicated.

## Future approaches

Given that osteosarcopenia is a newly established syndrome, its biological etiology and impact on clinical outcomes in older adults have only just begun to emerge. In this sense, further work is needed to advance knowledge on

- the temporal order of osteoporosis/osteopenia and sarcopenia leading to osteosarcopenia (epidemiological studies examining a life course approach may be required to answer this question);
- the biological mechanisms underpinning osteosarcopenia (the mechanism by which resistance exercise increases muscle and bone mass may provide further insight);
- the most accurate and practicable method for quantifying muscle mass in clinical and research settings (the emergence of the D_3_‐creatine dilution method has shown promise but requires further research);
- a biomarker for osteosarcopenia with high diagnostic value; and
- the synergistic effects of exercise training, nutritional interventions, and drug compounds in osteosarcopenic individuals.

## Summary

Osteosarcopenia is at the forefront of geriatric medicine and has received an upsurge of research in recent times. A myriad of lifestyle factors (sedentarism, obesity, and poor nutrition) interact via genetic, mechanical, and endocrine factors, which lead to muscle and bone loss and weakness, termed osteosarcopenia. Combined resistance and balance exercises with nutritional supplementation (whey protein, vitamin D, calcium, creatine) for those with deficiencies is a potent strategy to curb osteosarcopenia. Notwithstanding, further RCTs are needed in osteosarcopenic individuals. Regarding pharmacotherapies, denosumab may confer dual benefits on the muscle bone unit; however, further double‐blind RCTs are needed. Identifying these factors may aid in developing translational approaches to improving clinical practice, including diagnosis and treatment, and thus combat the growing burden of osteosarcopenia.

## Conflict of interest

The authors have no conflict of interest regarding this work.
